# Optimization of Ultrasound-Assisted Extraction of Polyphenols from *Myrtus communis* L. Pericarp

**DOI:** 10.3390/antiox8070205

**Published:** 2019-07-02

**Authors:** Nadia Bouaoudia-Madi, Lila Boulekbache-Makhlouf, Khodir Madani, Artur M.S. Silva, Sofiane Dairi, Sonia Oukhmanou–Bensidhoum, Susana M. Cardoso

**Affiliations:** 1 Laboratoire de Biomathématiques, Biophysique, Biochimie, et Scientométrie (L3BS), Faculté des Sciences de la Natureet de la Vie, Université de Bejaia, 06000 Bejaia, Algeria; 2 QOPNA & LAQV-REQUIMTE, Department of Chemistry, University of Aveiro, 3810-193 Aveiro, Portugal; 3 Faculté des Sciences de la Nature et de la Vie, Université de Jijel, 18000 Jijel, Algeria

**Keywords:** myrtle, ultrasound-assisted extraction, phenolic compounds, antioxidant capacity, liquid chromatography analysis, mass spectrometry

## Abstract

Response surface methodology (RSM) was used to optimize the extraction of phenolics from pericap of *Myrtus communis* using ultrasound-assisted extraction (UAE). The results were compared with those obtained by microwave-assisted extraction (MAE) and conventional solvent extraction (CSE) methods. The individual compounds of the optimized extract obtained by UAE were identified by ultra-high-performance liquid chromatography coupled with diode array detection and electrospray ionization mass spectrometry (UHPLC-DAD-ESI-MS^n^). The yield of total phenolic compounds (TPC) was affected more significantly by ethanol concentration, irradiation time, liquid solvent-to-solid ratio (*p* < 0.0001) and amplitude (*p* = 0.0421) and optimal parameters conditions set by the RSM model were 70% (*v*/*v*), 7.5 min and 30%, respectively. The experimental yield of TPC (241.66 ± 12.77 mg gallic acid equivalent/g dry weight) confirmed the predicted value (235.52 ± 9.9 mg gallic acid equivalent/g dry weight), allowing also to confirm the model validity. Under optimized conditions, UAE was more efficient than MAE and CSE in extracting antioxidants, which comprised mostly myricetin glycosides. Globally, the present work demonstrated that, compared to MAE and CSE, UAE is an efficient method for phenolic extraction from *M. communis* pericarp, enabling to reduce the working time and the solvent consumption.

## 1. Introduction

The *Myrtus* genus, belonging to the Myrtaceae family, comprises about 50 species that are native of the Mediterranean basin. Among those, *M. communis* is an aromatic evergreen perennial sub-shrub (high: 1–3 m) with white flowers (blossoming time: June to July) and dark blue ripe berries [1]. It is native to Southern Europe, North Africa and West Asia and widespread in the Mediterranean region. Its fruits are consumed either raw or processed in diverse products such as canned fruits, yogurts, beverages, jams and jellies. In addition, there has been a growing interest in the use of berry extracts as ingredients in functional foods and dietary [2]. Volatile oils, tannins, anthocyanins, fatty acids, sugars, and organic acids such as citric and malic acids are important components of these fruits [3]. In general, myrtle berries are accepted as being rich in phenolic compounds, which in turn are associated to the fruits claimed health effects, including the prevention of degenerative diseases, such as cancer and cardiovascular diseases [4]. In fact, phenolic compounds are accepted as potent antioxidants due to their double bonds and hydroxyl groups, being capable of preventing the oxidation of free radicals that may damage physiological molecules cells, such as lipid proteins and DNA [5]. Many studies have shown a positive relationship between the phenolics content and the antioxidant capacity of fruits and vegetables [4,5,6]. Moreover, the regular consumption of fruits and vegetables is believed to prevent oxidative stress events and oxidative-stress related diseases [7,8,9].

Due to countless beneficial characteristics of phenolic compounds in human health, research has been intensified, aiming to find fruits, vegetables, plants, agricultural and agroindustrial residues as sources of these bioactive components. Obtaining such compounds often requires many long and costly steps, such as extraction, isolation and identification [5], and often result in thermal degradation of various bioactive constituents [6,7]. In this context, the development of new extraction methods is one of the major challenges in technological innovation towards the direction of “Green chemistry” [8,9]. Among them, ultrasound-assisted extraction (UAE) and microwave-assisted extraction (MAE) are particularly attractive because of their simplicity, low cost of equipment, efficiency in extracting analytes from different matrices and the requirement of low energy, reduced quantity of solvent and/or time consumption [10], compared to conventional extraction methods, which have several disadvantages, such as the use of volatile and hazardous solvents, the long extraction time and more recovery energy [11]. The enhancement of the extraction process by ultrasounds is attributed to the disruption of the cell walls, reduction of the particle size and the increased mass transfer of the cell content to the solvent, caused by the collapse of the bubbles produced by acoustic cavitation [8]. The processing parameters optimization and interpretation of experiments compared to others has been previously done through response surface methodology (RSM) [12]. This latter has been shown to be a powerful tool in optimizing experimental conditions (factors) to maximize the response. With the experimental results of a response surface design, a mathematical polynomial model, describing the relation between a response (dependent variable) and the considered factors (independent variable), is built. The mathematical model, usually a second-order polynomial model, can be visualized graphically by drawing 2D contour plots or 3D response surface plots [13]. The model allows determining the optimum value of the independent variables (*X_i_*), as well as those of the dependent ones (*Y*).

Previous studies focusing on phenolic compounds and/or the antioxidant abilities of myrtle pericarp have been performed with extracts obtained by conventional methods [14,15,16], while, to our knowledge, there is no available information on the optimization of ultrasonic procedure for the extraction of phenolic compounds from this matrix, using a safer solvent such as ethanol, which is an organic solvent used in the food and pharmaceutical industries [17]. Therefore, the present study aimed at the optimization of UAE process parameters using RSM, including ethanol concentration, extraction time, irradiation amplitude and liquid-to-solid ratio, to maximize the content of the extracted phenolics. Levels of phenolic compounds and the antioxidant activity of pericarp *M. communis* extract obtained under the optimum setting parameters (UAE-OPT extract) were compared with those of extracts obtained by microwave-assisted extraction (MAE) and conventional solvent extraction (CSE) methods, using previously established conditions [18]. Then, the individual phenolic compounds present in the optimized extract obtained by UAE were identified by UHPLC-DAD-ESI-MS^n^.

## 2. Materials and Methods

### 2.1. Plant Material

The fruits of *M. communis* were harvested from spontaneous plants in Adakar, Bejaia, located in the northeast of Algeria. The collected samples were identified by the Vegetable Ecological Laboratory of the Algiers University, Algeria and a voucher specimen was deposited at the Herbarium of Natural History Museum of Aix-en-Provence, France, under the voucher number D-PH-2013-37-12. Berries were washed and then dried in a static oven at 40 °C for one week. Pericarps were separated manually from seeds and further grounded in an electrical grinder (A11Basic, IKA, Retsch, Germany), which was then sieved to obtain a fine powder (<250 µm).

### 2.2. Extraction of Phenolic Compounds

#### 2.2.1. Ultrasound Extraction

UAE was performed in an ultrasonic apparatus (Vibra cell, VCX 75115 PB, SERIAL No. 2012010971 MODEL CV 334, SONICS, Newtown, Connecticut, USA) with a working frequency fixed at 20 kHz. For extraction, 1 g of the pericarp powder was placed in a 250 mL amber glass bottle containing ethanol. The suspension was exposed to acoustic waves under distinct setting parameters (solvent concentration, irradiation time, ultrasound amplitude and solvent-to-solid ratio). The temperature was maintained constant by circulating external cold water and checking the temperature using a T-type thermocouple [5]. Indeed, ultrasound is considered a non-thermal technology, since it increases only the local temperature without affecting the surrounding environment [19]. After extraction, the solution was filtered through a sintered glass filter of porosity 2.

To determine the effect of ethanol concentration, irradiation time, ultrasound amplitude and solvent-to-solid ratio on the extraction yield of phenolic compounds from myrtle pericarp, RSM was applied with a Box–Behnken Design (BBD) [5]. This design resulted in the testing of four factors in a single block of 30 sets of test conditions (Table 1). The constant values for irradiation time, liquid-to-solid ratio and ethanol concentration in the UAE trials were 10 min, 50 mL/g and 50% (*v*/*v*), respectively.

(1) $$Y=\beta_{0}+\sum_{i=0}^{k}\beta_{i}X_{i}+\sum_{i=1}^{k}\beta_{ii}X^{2}+\sum_{i>1}^{k}\beta_{ij}X_{i}X_{j}+E$$

where *X_i_*, *X_j_*, …, *X_k_* are the independent variables affecting the responses *Y* (the yield of total phenolic compounds); *β_i_*, *β_ii_* and *β_ij_* are the regression coefficients for linear, quadratic and interaction terms, respectively; and k is the number of variables.

The factor levels were coded as −1 (low), 0 (central point or middle) and 1 (high), respectively, according to Equation (2):

*X_i_* = (*X_i_* − *X_0_*)/Δ*X*… (2)

where *X_i_* is the coded value of the variable *X_i_*; *X_0_* is the value of *X* at the center point; and Δ*X* is the step change.

#### 2.2.2. Microwave-Assisted Extraction

Phenolic extracts were obtained using a domestic microwave oven (Samsung MW813ST, Kuala Lumpur, Malaysia) adapted by adding of a condenser [18]. The apparatus operated at a frequency of 2450 MHz and a maximum output power of 1000 W with a 100 W increment. The size of the heating cavity was 37.5 cm (L) × 22.5 cm (W) × 38.6 cm (D). The applied extraction conditions corresponded to those previously optimized [18]. A volume of 32 mL of 42% ethanol concentration was added to 1 g of pericarp *Myrtus* powder in a flat-bottomed flask. The mixture was irradiated at 500 W for 62 s. The resultant extract was then filtered through a sintered glass filter of porosity 2 and was stored at 4 °C until further analysis.

#### 2.2.3. Conventional Solvent Extraction

Conventional solvent extract followed the procedure established by Dahmoune et al. [5]. One gram of myrtle powder was placed in a conical flask, and 50 mL of 50% (*v*/*v*) ethanol were added. After stirring for 2 h, the mixture was filtered through a sintered glass filter of porosity 2 and the extract was stored at 4 °C until further use.

### 2.3. Analytical Determinations

#### 2.3.1. Total Phenolic and Flavonoid Contents

The total phenolic content (TPC) of the UAE, MAE and CSE extracts was assessed according to the method of George et al. [20] and expressed as mg of gallic acid equivalent (GAE) per gram of myrtle pericarp powder on dry weight (DW) basis (mg GAE g^–1^ DW). The total flavonoid content was estimated by the aluminum trichloride method according to Quettier-Deleu et al. [21] and the results were expressed as mg of quercetin equivalent per g of myrtle pericarp powder, on a DW basis.

#### 2.3.2. Total Monomeric Anthocyanins and Condensed Tannin Contents

Total monomeric anthocyanin content was determined by the pH-differential method [22], and the results were expressed as mg cyanidin-3-*O*-glucoside equivalents per g of myrtle pericarp powder on a DW basis. The condensed tannin content was determined by the HCl–vanillin method as described by Aidi Wannes et al. [23] and the results were expressed as mg catechin equivalents per g of myrtle pericarp powder on DW basis.

#### 2.3.3. Antioxidant Activity

The antioxidant activity of all samples was tested by using two different tests, namely 1,1-diphenyl-2-picrylhydrazyl radical (DPPH**^●^**) scavenging activity and reducing power methods [24]. DPPH**^●^** solution (60 µM) was prepared in absolute methanol and reaction was performed by the adding of 3 mL of this solution to 1 mL of the extracts, during 20 min at 37 °C in the dark. Thereafter, the absorbance was measured at 515 nm. The inhibition rate of the extracts was calculated according Equation (3).

(3) $$\%\;Scavenging=\frac{\left(A_{control}-A_{extract}\right)}{A_{control}}\times 100$$

where *A_control_* is the absorbance of DPPH**^●^** and distilled water *A_sample_* is the absorbance of DPPH**^●^** and sample extract. α-tocopherol and BHA (250 µg/mL) were used as positive controls.

For reducing power assay, 1 mL of desired dilution was mixed with 2.5 mL of sodium phosphate buffer (0.2 M, pH 6.6) and 2.5 mL of 1% (*m*/*v*) potassium ferricyanide K_3_[Fe(CN)_6_)], followed by incubation in a water bath at 50 °C for 20 min and the addition of 2.5 mL of 10% (*m*/*v*) trichloroacetic acid. At last, an aliquot of the resulting solution (1 mL) was added to 5 mL of distilled water and 1 mL of 0.1% (*m*/*v*) of FeCl3·6H2O. Note that this method estimates the ability to reduce Fe^3+^ to Fe^2+^. Antioxidant compounds present in the samples form a colored complex with potassium ferricyanide, trichloroacetic acid and ferric chloride, which is measured at 700 nm.

### 2.4. Identification of Phenolic Compounds by UHPLC-DAD-ESI-MS^n^

The phenolic compounds of the UAE-OPT extract were characterized by UHPLC-DAD-ESI-MS^n^ (DAD: diode array detector; ESI: electrospray ionization) analysis on an Ultimate 3000 (Dionex Co., USA) apparatus equipped with an ultimate 3000 Diode Array Detector (Dionex Co., San Jose, CA, USA) and coupled to a mass spectrometer. Analysis was run on a Hypersil Gold (Thermo Scientific, San Jose, CA, USA) C18 column (100 mm length; 2.1 mm i.d.; 1.9 μm particle diameter, end-capped) and its temperature was maintained at 30 °C. The mobile phase was composed of (A) 0.1% of formic acid (*v*/*v*) and acetonitrile (B). The solvent gradient started with 5% of Solvent B, reaching 40% at 14 min and 100% at 16 min, followed by the return to the initial conditions. The flow rate was 0.1 mL min^–1^ and UV–Vis spectral data for all peaks were accumulated in the range 200–700 nm while the chromatographic profiles were recorded at 280, 340 and 530 nm.

The mass spectrometer consisted of a Thermo LTQ XL (Thermo Scientific, San Jose, CA, USA) ion trap MS (mass spectrometer) apparatus equipped with an ESI source, operating in negative and positive modes, under the pre-established conditions [25].

### 2.5. Statistical Analysis

Each extraction trial and all the analyses were carried out in three independent analysis performed in triplicate. The influence of individual factors on the TPC yield (single-factor experiment) was estimated by Analysis of Variance (ANOVA) and Tukey’s post hoc test with a 95% confidence level, while data obtained from the BBD and Central Composite Design (CCRD) trials were analyzed through ANOVA for the response variable to evaluate the model significance and suitability. Significant and highly significant levels were set for *p* < 0.05 and *p* < 0.01, respectively. The John’s MacIntosh Product (Version 7.0, SAS, Cary, NC, USA) and Design-Expert (Trial version 10.0, SAS, Cary, NC, USA) software packages were used to construct the BBD and CCRD and to analyze all the results. Principal Component Analysis (PCA) was applied to detect the relationships between contents of phenolic compounds, flavonoids, anthocyanins, tannins, as well as antioxidant activity and their extraction methodologies i.e., UAE, MAE and CSE. All tests were done in triplicate.

## 3. Results and Discussion

### 3.1. Optimization of UAE Conditions

#### 3.1.1. Modeling and Fitting the Model Using RSM

The experimental design and subsequent response allied to TPC are summarized in Table 1, with results from TPC recovery varying in the range of 79–235 mg GAE/g DW.

The least square technique was used to calculate the regression coefficients of the intercept, linear, quadratic, and interaction terms [26] (Table 2). Notably, the linear parameters, namely ethanol concentration, irradiation time and liquid–solid ratio (*p* < 0.0001), followed by amplitude (*p* = 0.0421) significantly affected the extraction content of phenolic compounds. The quadratic terms *X_2_*^2^ and *X_4_*^2^ were highly significant at the level *p* < 0.001, while the *X_1_*^2^ and *X_3_*^2^ terms were insignificant (*p* > 0.05). Regarding TPC yield, the interaction of ethanol concentration with amplitude of ultrasound (*X_1_*–*X_3_*) and with liquid to solid ratio (*X_1_*–*X_4_*), and that of irradiation time amplitude of ultrasound (*X_2_*–*X_3_*) were highly significant (*p* < 0.0001), followed by irradiation time with liquid-to-solid ratio (*p* = 0.0054), amplitude of ultrasound with liquid-to-solid ratio (*p* < 0.0094) and ethanol concentration with irradiation time (*p* = 0.0367). Those significant terms played a dominant role in myrtle pericarp extraction by ultrasound. Indeed, those significant terms played a dominant role in myrtle pericarp extraction by ultrasound. This is justified by the analyses of variance, as represented in Table 2, with significant *p*-values (*p* < 0.0001) for the linear parameters, namely ethanol concentration (*X_1_*), irradiation time (*X_2_*) and liquid–solid ratio (*X_4_*), the quadratic terms *X_2_*^2^ and *X_4_*^2^, and the interaction terms (*X_1_*–*X_3_*), (*X_1_*–*X_4_*) and (*X_2_*–*X_3_*). However, insignificant *p*-values (*p* > 0.0001) were obtained for the third linear parameter, amplitude (*X_3_*), the quadratic terms *X_1_*^2^ and *X_3_*^2^ and the interaction terms (*X_1_*–*X_2_*), (*X_2_*–*X_4_*) and (*X_3_*–*X_4_*).

Based on the significant terms, the regression equation for the UAE efficiency was obtained as follows:

*Y* = 205.032 + 10.998*X_1_* + 14.043*X_2_* − 3.994*X_3_* + 27.390*X_4_* + 5.049*X_1_X_2_* − 11.460*X_1_X_3_* − 10.588*X_1_X_4_* + 15.196*X_2_X_3_* − 7.198*X_2_X_4_* − 6.580*X_3_X_4_* − 1.526*X_1_*^2^ − 11.561*X_2_*^2^ − 0.888*X_3_*^2^ − 17.876*X_4_*^2^ (4)

Note that the *p*-value can be employed to check the interaction strength between independent factors. From this analysis, *p*-value <0.0001 indicated that the response surface quadratic model was significant, which means that the model represented the data satisfactorily. The adjusted coefficient of determination *(R*^2^adj) and the coefficient of determination (R^2^) were 0.9553 and 0.9776, respectively, which implied that the sample variations of 97.76% for the UAE efficiency of myrtle pericarp phenols were attributed to the independent variables, and only 2.24% of the total variations could not be explained by the model, indicating a good degree of correlation between experimental and predict values of the TPC yield. In addition, the low value of coefficient of variance (3.71%) clearly indicated that the model was reproducible and reliable [27]. All these results indicate that the model could work well for the prediction of TPC in the myrtle pericarp extracts.

#### 3.1.2. Response Surface Analysis (RSA)

**Figure 1:**
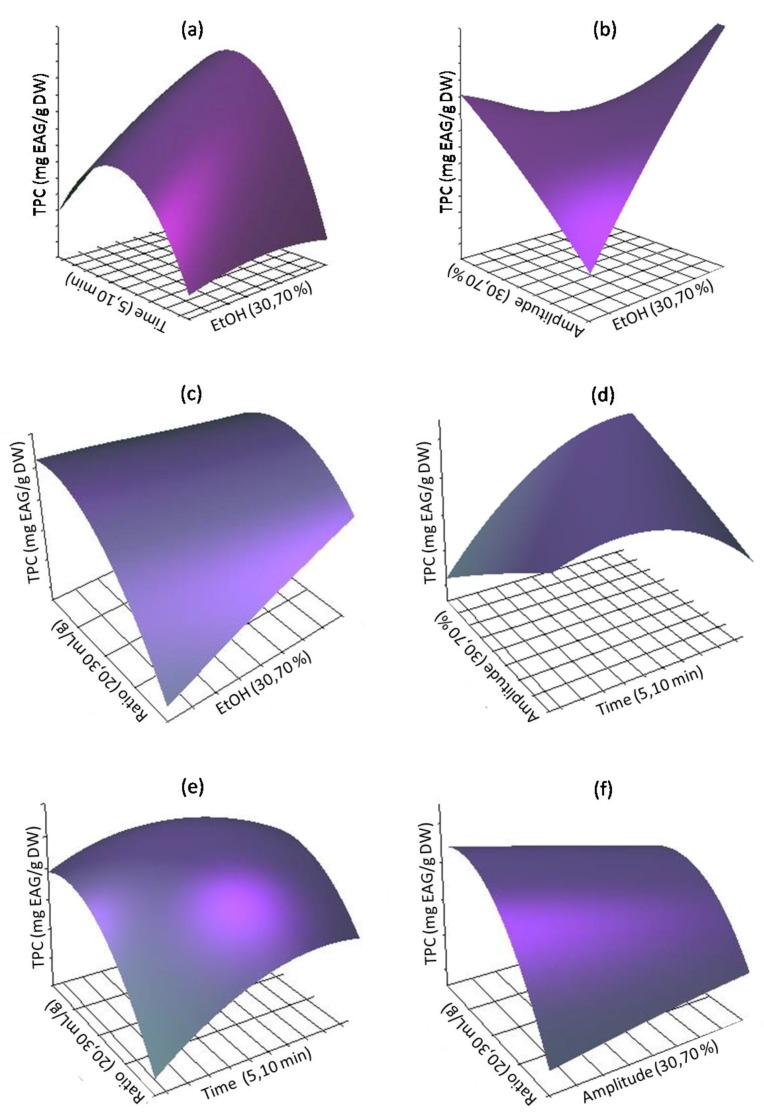
Response surface analysis for the Total phenolic compounds (TPC) with UAE with respect to: (**a**) ethanol concentration and irradiation time; (**b**) ethanol concentration and amplitude; (**c**) ethanol concentration and solvent-to-solid ratio; (**d**) extraction time and amplitude; (**e**) extraction time and solvent-to-solid ratio; and (**f**) amplitude and solvent-to-solid ratio.

To provide a better understanding of the interaction between factors, the 3D response surface plot was constructed (Figure 1) using Equation (4). The graphs were generated by plotting the response using the z-axis against two independent variables, while keeping the other independent variable at the fixed level. Figure 1A–C shows the interactions between the ethanol concentration and each of the three other factors, namely irradiation time, amplitude and liquid-to-solid ratio, respectively, on the recovery of TPC. As shown, an increase of ethanol concentration from 20% to 80% (*v*/*v*), or extraction time from 5 to 10 min resulted in a rapid enhancement of TPC with a maximum of 235.21 mg GAE/g being recovered with an irradiation time of 7.5 min and ethanol concentration of 70% (*v*/*v*).

The high phenolic content indicates that the mixture ethanol/water at 70% (*v*/*v*) allowed the solubilization of phenolics from *M. communis* pericarp, thus confirming the results of the single factor experiments [28] that explained the efficiency of the ultrasonic method by the fact that sonication improved the hydration and fragmentation process and hence facilitates the mass transfer of solutes to the extraction solvent. For the extraction yield of TPC performed at fixed extraction time and liquid-to-solid ratio, with varying ethanol concentration and amplitude (Figure 1B), it was possible to conclude that maximum recovery (210.05 mg GAE/g) was achieved for 70% (*v*:*v*) of ethanol and an ultrasound amplitude of 35%. This fact can be explained by the larger amplitude ultrasonic wave that promotes the liquid medium to produce more cavitation bubbles, thus resulting in a stronger pressure, capable of destroying the cell wall and accelerating mass transfer [29]. Figure 1C shows an enhancement of TPC that reached a peak value of 230.15 mg GAE/g, for 70% (*v*:*v*) ethanol and about 30 mL/g of liquid-to-solid ratio. A higher ratio corresponds to a greater concentration difference between the exterior solvent and the interior tissues of *Myrtus* pericarp. It prominently prompted the TPC to be rapidly dissolved, which resulted in an increase in the extraction yield. The response surface plot for the significative interactive effect of irradiation time and amplitude of ultrasound on the response value at a fixed ethanol concentration and liquid-to–solid ratio is shown in Figure 1D. A higher TPC was obtained with the irradiation time at 10 min and amplitude of 30%; these results confirm those reported in the literature [30,31]. Figure 1E shows an interaction between extraction time and the liquid-to-solid ratio (*p* < 0.05). The best content (148 mg GAE/g) was found with the solid–liquid ratio of about 30 mL/g and the radiation time of 10 min. The increase of the ethanol proportion required high sonication intensity to generate the cavitation bubbles. However, a higher increase in the liquid-to-solid ratio diminished the supply of ultrasonic energy density and negatively affected the extraction yield.

The yield of TPC constantly improved with the increase of both amplitude of ultrasound and liquid-to-solid ratio, reaching a maximum when *X_3_* and *X*_4_ became 32% and 20% (*v*/*v*), respectively (Figure 1F). Beyond this level, the yield of TPC reduced with the increase of *X*_1_ and *X*_4_. Hence, the interactive effect of *X_3_* and *X_4_* was remarkable. Overall, these results indicate that the TPC extraction yield was more significantly affected (*p* < 0.0001) by linear parameters, namely ethanol concentration, irradiation time and liquid-to-solid ratio.

#### 3.1.3. Validation and Verification of the Predictive Model

According to the result of response surface and prediction by this built model, the optimal conditions were thus obtained for the following conditions: ethanol at 70% (*v*/*v*), 7.5 min extraction time, 30% amplitude and a liquid-to-solid ratio of 28 mL/g. To ensure that the predicted result was not biased to the practical value, experimental rechecking was performed using these deduced optimal conditions. The predicted extraction yield of TPC in UAE-OPT was 235.52 ± 9.9 mg GAE/g, that was consistent with the experimental yield of 241.66 ± 12.77 mg GAE/g DW (Table 3). The results showed no significant difference between the experimental and the predicted values. This strong correlation between experimental and the predicted values indicates that the response of regression model is adequate to reflect the expected optimization for the extraction of antioxidants from *M. communis* pericarp.

### 3.2. Comparison between UAE, MAE and CSE Methods

Remarkably, the highest TPC was obtained by UAE (241.60 ± 12.77 mg GAE/g). This corresponded to four and three times higher than that obtained by MAE and CSE, respectively, thus indicating that the application of UAE has a positive effect on the extraction of TPC (Table 3). The highest levels of TPC in UAE-OPT extract was reflected by its higher amounts of flavonoids, anthocyanins and tannins (18.99 ± 1.31 mg QE/g; 25.06 ± 0.36 mg/g; 35.56 ± 0.36 mg CE/g, respectively). These findings are consistent with those reported in the literature [32] and are mainly attributed to the fact that ultrasound radiation can facilitate mass transfer and accelerate the extracting process so that the extraction of bioactive compounds may be improved. Hence, according to the overall data, it is possible to conclude that the herein optimized UAE process yields higher levels of bioactive compounds in a short time and requires less solvent consumption than MAE and CSE. Note that, in this study, the operating temperature in the UAE-OPT was kept constant at room temperature, excluding any heating effect. This might positively or negatively influence the polyphenols recovery depending on the applied amplitude.

**Figure 2:**
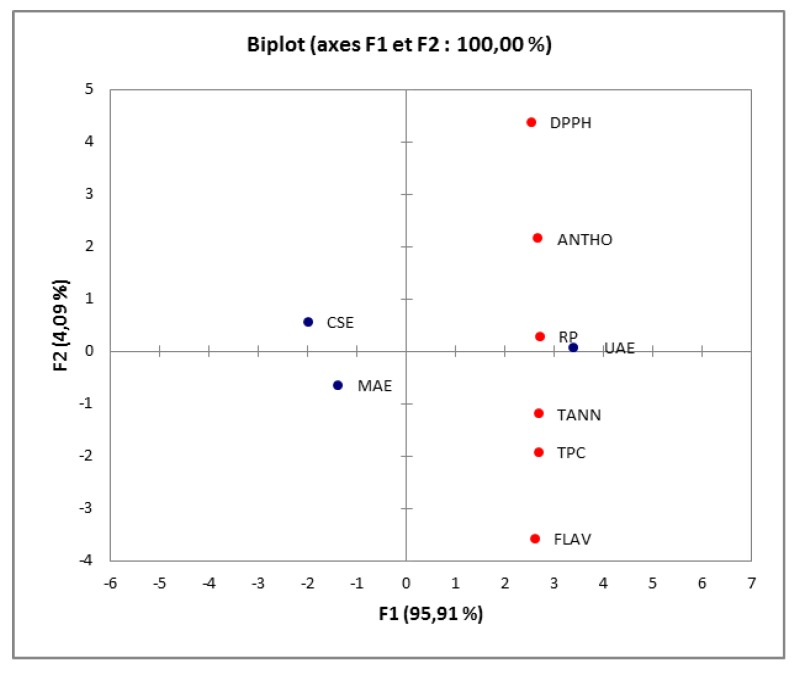
Principal component analysis of phenolic compounds for *M. communis* pericarp with UAE, MAE and CSE. FLAV, flavonoids; ANTHO, anthocyanins; TANN, tannins.

The antioxidant capacity of the extracts was assessed by DPPH**^●^** scavenging and ferric reducing antioxidant power assays. The results show that UAE-OPT extract presented higher DPPH**^●^** scavenging ability (90.71% inhibition) when compared to CSE (88.03% inhibition) and MAE (87.16% inhibition) extracts. The same tendency was also observed for reducing power, since the absorbance at 700 nm for UAE-OPT extract was considerably higher than those obtained for MAE and CSE (0.439 ± 0.006 and 0.429 ± 0.01, respectively). This means that UAE method is more efficient for the recovery of antioxidants than the herein tested microwave and conventional solvent extraction methods, a fact that is probably due to its superior richness in phenolic components, including flavonols [33] and as evidenced in the following section. This information was also confirmed by PCA analysis. PCA was applied to the extracts (UAE-OPT, MAE and SCE) for phenolic compounds (TPC, flavonoids, anthocyanins and tannins) and antioxidant activity, where the two chosen factors justified 100.0% of total variance. The resulting plots allowed selecting the better extraction method of different compounds of myrtle pericarp, and clearly divided the samples into three groups, depending on the extraction method (Figure 2). For PC1, which explains 95.91% of the total variance, the first group showed a positive correlation with PC1, thus confirming that UAE was the best extraction method for phenolic compounds with potent antioxidant activity. The highest correlation was found between antioxidant activity (DPPH^•^ and RP essay) and anthocyanins, hence suggesting that these compounds might have a key influence on the antioxidant capacity of the extracts. The best correlation between the MAE and TPC, flavonoids and tannins were observed in the second group. PC2 explains only better 4.09% of the experimental variability, which could essentially be associated to the CSE method and anthocyanins content (the third group).

### 3.3. Identification of Phenolics by UHPLC-DAD-ESI-MS^n^ Analysis

**Figure 3:**
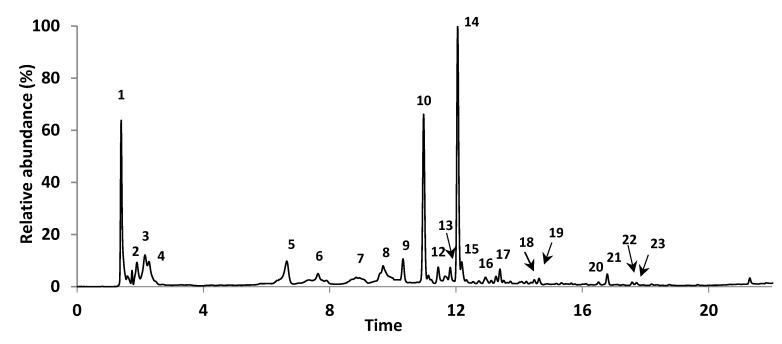
Chromatographic profile at 280 nm of *M. communis* pericarp extract obtained by UAE extraction at optimized conditions. Numbers in the figure correspond to the eluted UHPLC peaks for which UV and MS data are summarized in Table 4.

The UAE-OPT extract was analyzed by UHPLC-DAD-ESI-MS^n^ to further elucidate its phenolic profile. The registered chromatogram at 280 nm is shown in Figure 3 and the UV-Vis and MS^n^ spectral data of eluted peaks are summarized in Table 4.

Among the distinct phenolic groups found in the extract, flavonols were the prevalent components. Overall, eleven flavonol glycosides were detected, being myricetin glycosides, namely myricetin-*O*-hexoside and myricetin-*O*-deoxyhexoside (eluted in Peaks 10/11 and 14/15, respectively) the major abundant ones, which probably correspond to myricetin-3-*O*-galactoside and myricetin-3-*O*-rhamnoside, since these are known to be present as main phenolic components in distinct organs of *M. communis* plant [34,35,36,37,38].

Besides the above compounds, four other myricetin glycosides were found in the extract. The compound eluted in Peak 9, showing a [M-H]^−^ at *m/z* 631, corresponded to myricetin-*O*-galloyl-hexoside, since the main fragments in MS^2^ spectrum were formed by the loss of 152 Da (equivalent to a galloyl moiety) and 332 Da (equivalent to the simultaneous loss of galloyl and hexosyl units). This could possibly correspond to myricetin 3-(6’’-*O*-galloyl galactoside), which has been previously reported in leaves [32,34,36,38] and berries [16]. In addition, the compounds with [M-H]^−^ at *m/z* 449 (Peak 13) and at *m/z* 625 (co-eluted in Peak 18) were, respectively, assigned to myricetin-*O*-pentoside and myricetin-*O*-hexosyl-deoxyhexoside, according to their fragmentation pattern, which showed the loss of a pentosyl (132 Da) and deoxyhexosyl plus hexosyl (308 Da) moieties, respectively. In turn, the compound eluted in Peak 19 at 14.6 min with a pseudomolecular ion at *m/z* 569 and fragment ions at *m/z* 485 (equivalent to galloyl ester moiety) and 317 (myricetin) was tentatively assigned to a galloylester of myricetin.

The three remaining flavonols detected in the UAE-OPT extract were assigned to quercetin and kaempferol derivatives. From those, the compound eluted in Peak 12 was characterized by a [M-H]^−^ at *m/z* 615 and fragment ions at *m/z* 463 (−152 Da, loss of galloyl group) and 301 (−162 Da, loss of an hexosyl group), and was tentatively assigned to quercetin-*O*-hexoside-gallate on the basis of data reported in the literature [39,40,41]. This compound has been already reported in Myrtaceae family, namely in *Eucalyptus* species [42,43,44] and two other species from the same family, namely *Myrcia multiflora* extracts [45] and *Eugenia edulis* [46]. In addition, the compound eluted in Peak 17 with a deprotonated ion at *m/z* 447 and a base peak fragment ion at *m/z* 301 (−146, equivalent to the loss of a deoxyhexose unit), was identified as quercetin-*O*-deoxyhexoside according to literature data, probably corresponding to quercetin-3-*O*-rhamnoside [39,40]. This last flavonoid was previously detected in pericarp [29], berries [31,47] and leaves [30,31,35] of *M. communis*. Finally, the flavonol eluted in Peak 16 ([M-H]^−^ at *m/z* 447) presented the main fragment ion at *m/z* 285 in the MS^2^ spectrum, which in turn showed a fragmentation pattern coherent with kaempferol. Based on UV-Vis spectra (UV_max_ at 265 and 353) and MS^n^ spectral data, this compound was assigned to kaempferol-*O*-hexoside.

Besides flavonols, other flavonoids in UAE-OPT extract corresponded to anthocyanins that were eluted from 7.6 min to 9.7 min (Peaks 6–8). Note that, in general, anthocyanins are preferred detected as [M]^+^ in ESI in the positive mode, while typically they show [M-2H]^−^ in the negative mode [47], as represented in Table 4. Overall, according to UV-Vis and MS^n^ spectral data, these compounds were assigned to delphinidin, petunin and malvidin derivatives. In more detail, the compound in Peak 6 exhibiting a [M-2H]^−^ at *m/z* 463 and a base peak MS^2^ fragment ion at *m/z* 301 (−162 Da) was assigned to delphinidin-*O*-hexoside by comparison with data reported in the literature [48,49,50]. In turn, petunidin-*O*-hexoside and a petunidin-*O*-hexoside derivative were eluted in Peaks 7 and 8, respectively. The first showed a [M-2H]^−^ at *m/z* 477 and a main MS^2^ fragment ions at *m/z* 315/314 [48,49,50] while ions corresponding to petunidin-*O*-hexoside and its hydrated form (at *m/z* 477 and *m/z* 495, respectively) were predominant in MS^2^ spectrum of the latter compound. The petunidin-*O*-hexoside derivative was co-eluted with malvidin-*O*-hexoside ([M-2H]^−^ at *m/z* 477→329). Note that, except for petunidin-*O*-hexoside, hexosides of delphinidin, petunin and malvidin have already been described in distinct organs of *M. communis*, including pericarp [14,30,31,51,52].

Several non-flavonoid compounds could also be observed in UEA-OPT extract, including caffeoyl hexoside, gallic acid and galloyl derivatives. The first ([M-H]^−^ at *m/z* 341→179, eluted in Peak 1) was the only hydroxycinnamic acid found in the extract. Gallic acid ([M-H]^−^ at *m/z* 169→125, eluted in Peak 4), has been described in the literature for extracts obtained from the pericarp [16] berries [16,30] and leaves [38,52].

Regarding galloyl derivatives (typical UV_max_ at 273–276 nm), these enclosed esters of mono- or di-galloyl groups with a hexose or quinic acid unit, or even with myrtucommulone-type groups. In detail, the compound eluted in Peak 2 with a [M-H]^−^ at *m/z* 331 and corresponding fragments at *m/z* 271, 169, 241, 211, 193 and 125, was assigned to a galloyl hexoside [53], presumably galloyl-3-*O-β*-D-galactoside-6-*O*-gallate, since this latter has been previously reported in *M. communis* leaves [2,38]. Besides, two isomers of galloyl quinic acid ([M-H]^−^ at *m/z* 343→191, 169, 125) could be found in Peaks 3 and 5, while a digalloyl hexoside ([M-H]^−^ at *m/z* 483→271, 331, 313, 439, 193, 169) and digalloyl quinic acid ([M-H]^−^ at *m/z* 495→343, 325, 191, 169) were detected as co-eluted compounds in Peak 6. All these galloyl derivatives have been previously detected in *M. communis* leaves [35,38,52]. 

Moreover, four gallomyrtucommulone-type derivatives were found in UAE-OPT extract. All these compounds showed a UV_max_ at 276 nm, and similar fragment ions in MS^n^ spectra, including ions at *m/z* 331, 313, 271 and 211, which are typically formed in galloylhexoside [43]. Indeed, the ion at *m/z* 331 correspond to the galloyl hexoside moiety, while ions at *m/z* 271 and *m/z* 211 result from the cross-ring fragmentation of the hexose unit in the galloyl hexoside moiety and that at *m/z* 313 can be formed due to the loss of water molecule from the latter. Among these compounds, those eluted in Peaks 20 and 21 ([M-H]^−^ at *m/z* 583 and 567, respectively) were assigned to gallomyrtucommulone F and gallomyrtucommulone C, in accordance to previous data reported in *M. communis* leaves [36]. Besides these two compounds, the extract also contained two isomeric unidentified gallomyrtucommulone-type derivatives (MW 468 Da) that presumably vary in their acyl chain regarding those previously identified.

## 4. Conclusions

The response surface methodology was successfully employed to optimize total phenolic extraction yield from dried *M. communis* pericarp by non-conventional solvent extraction process, namely using UAE. As compared to MAE and CSE extractions, the proposed UAE method allowed a higher phenolic recovery yield and antioxidant activity with a short working time and a lower solvent consumption. The quantification of the amounts of phenolic compounds in the three types of extract complemented with PCA analysis also allowed concluding that the *M. communis* pericarp extract obtained under optimal UAE experimental conditions contained higher levels of flavonoids, tannins and anthocyanin than the remaining extracts and, particularly, the latter phenolic components could be correlated to its antioxidant activity. According to UHPLC-DAD-ESI-MS^n^ analysis, flavonols, particularly myricetin-*O*-hexoside and myricetin-*O*-deoxyhexoside, were the prevalent phenolic components of UAE-OPT.

## Figures and Tables

**Table 1:** Central composite design with the observed responses and predicted values for yield of total phenolic compounds of *Myrtus communis* pericarp using the UAE method.

Run	*X_1_*-Ethanol (%, *v*/*v*)	*X_2_*-Irradiation Time (min)	*X_3_*-Amplitude (%)	*X_4_*-Solvent-to Solid Ratio (mL/g)	TPC Recovery (mg GAE/g DW)
1	50	2.5	50	25	134.93 ± 11.37
*2*	70	10	70	30	195.24 ± 0.99
*3*	30	5	30	20	105.29 ± 11.72
4	70	5	30	30	221.73 ± 3.64
5	50	7.5	50	25	200.50 ± 12.82
6	50	7.5	50	15	78.90 ± 10.45
7	50	7.5	50	25	200.90 ± 28.02
8	50	7.5	50	25	200.10 ± 23.11
9	50	7.5	90	25	200.02 ± 13.23
10	70	10	30	30	214.07 ± 14.66
11	50	7.5	10	25	210.72 ± 2.70
12	50	7.5	50	25	210.21 ± 15.39
13	10	7.5	50	25	170.43 ± 9.38
14	30	10	70	20	159.56 ± 10.02
15	30	10	70	30	228.39 ± 12.96
16	50	7.5	50	25	203.34 ± 16.40
17	70	10	70	20	200.42 ± 14.47
18	70	10	30	20	185.51 ± 13.34
19	50	7.5	50	35	195.94 ± 12.80
20	50	12.5	50	25	190.43 ± 15.47
21	70	5	70	30	142.96 ± 9.60
22	30	10	30	20	118.92 ± 12.08
23	70	5	70	20	115.45 ± 16.12
24	30	5	30	30	219.12 ± 18.72
25	30	10	30	30	180.77 ± 9.38
26	70	5	30	20	161.68 ±9.42
27	30	5	70	30	179.22 ± 9.70
28	90	7.5	50	25	235.21 ±17.36
29	50	7.5	50	25	210.21 ± 16.60
30	30	5	70	20	111.38 ± 7.63

TPC results are expressed as means ± standard deviation; GAE, gallic acid equivalent; UAE, ultrasound-assisted extraction; DW, dry weight.

**Table 2:** Estimated regression coefficients for the quadratic polynomial model and analyzes of variance (ANOVA) for the experimental results.

Parameters	Estimated Coefficients	Standard Error	DF ^a^	Sum of Squares	F Ratio ^b^	Prob > F
**Model**			14	46971.996	43.8356	<0.0001
**Intercept**	205.032	3.912523			52.40	<0.0001
**Linear**						
*X_1_*-Ethanol	10.99875	1.736676	1	2903.340	37.9327	<0.0001
*X_2_*-Time	14.04375	1.736676	1	4733.446	61.8434	<0.0001
*X_3_*-Amplitude	−3.994583	1.736676	1	382.961	5.0035	0.0421
*X_4_*-Ratio	27.390417	1.736676	1	18005.638	235.2474	<0.0001
**Quadratic**						
*X_1_* ^2^	−1.526438	1.717541	1	60.454	0.7898	0.3892
*X_2_* ^2^	−11.56144	1.717541	1	3468.113	45.3116	<0.0001
*X_3_* ^2^	−0.888938	1.717541	1	20.503	0.2679	0.6128
*X_4_* ^2^	−17.87644	1.717541	1	8291.469	108.3279	<0.0001
**Interaction**						
*X_1_*–*X_2_*	5.049375	2.187167	1	407.939	5.3298	0.0367
*X_1_*–*X_3_*	−11.46062	2.187167	1	2101.535	27.4570	<0.0001
*X_1_*–*X_4_*	−12.58812	2.187167	1	2535.37	33.1252	<0.0001
*X_2_*–*X_3_*	15.196875	2.187167	1	3695.120	48.2775	<0.0001
*X_2_*–*X_4_*	−7.198125	2.187167	1	829.008	10.8312	0.0054
*X_3_*–*X_4_*	−6.580625	2.187167	1	692.874	9.0525	0.0094
**Lack of fit**			10	94.0987	4.550	0.0911
**Pure error**			4			
***R*^2^**					0.9776	
**Adjusted *R*^2^**					0.9553	
**C.V. %**	3.71%.					
**RMSE**	8.7186					
**CorTotal ^c^**			28	48043.545		

^a^ Degree of freedom; ^b^ the model mean square to error mean square ratio; ^c^ corrected total. DF, degree of freedom; F Ratio, freedom ratio; Prob, probability; C.V., coefficient of variation.

**Table 3:** Comparison of extraction yield of polyphenols obtained by optimized ultrasound-assisted (UAE-OPT), microwave-assisted (MAE) and conventional solvent (CSE) methods.

Method	EtOH (%)	Time (min)	US amp (w/power)	Liq:sol (mL/g)	TPC (mg GAE/g)	Flavonoids (mgQE/g)	Anthocyanin (mg/g)	Tannins (mg CE/g)	DPPH (%)	RP (Abs 700 nm)
**UAE-OPT**	70	7.5	30	28	241.66 ± 12.77 ^a^	18.99 ± 1.31 ^a^	25.06 ± 0.36 ^a^	35.56 ± 0.36 ^a^	90.71 ± 0.23 ^a^	0.568 ± 0.002 ^b^
**MAE**	42	62	500	32	119.59 ± 8.40 ^b^	11.5 ± 0.01 ^b^	5.64 ± 0.06 ^c^	31.70 ± 1.00 ^b^	87.16 ± 0.28 ^b^	0.439 ± 0.006 ^b^
**CSE**	50	7200		50	76.40 ± 7.27 ^c^	6.95 ± 0.20 ^c^	6.96 ± 0.72 ^b^	30.70 ± 0.17 ^c^	88.03 ± 1.04 ^b^	0.429 ± 0.001 ^b^
**BHA** **α-tocopherol**									26.98 ± 0.69 ^c^ 17.17 ± 0.4 ^d^	1.37 ± 0.03 ^a^ 0.53 ± 0.01 ^b^

Contents of TPC, flavonoids, anthocyans and tannins are means ± standard deviation. Different letters in the same row indicate significant differences (*p* < 0.05) according to the ANOVA test. BHA, butylated hydroxyanisole; CE, catechin equivalents; DPPH, 1,1-diphenyl-2-picrylhydrazyl radical; EtOH, ethanol; GAE, gallic acid equivalents; Liq:sol, liquid-to-solid ratio; QE, quercetin; RP, ferric reducing antioxidant power; US amp, ultrasound amplitude.

**Table 4:** UHPLC-DAD-ESI-MS^n^ data for *M. communis* pericarp extract obtained under optimized UAE conditions.

No. Peak	t_R_ (min)	λmax (nm)	(*m/z*)	MS^n^ Ions (*m/z*)	Probable Compound
1	1.4	275	191 ^a^	MS^2^[191]: 173, 127, 111, 93	Quinic acid
			341 ^a^	MS^2^[341]: 179	Caffeoyl-*O*-hexoside
2	1.9	276	331	MS^2^[331]: 271, 169, 241, 211, 193, 125; MS^3^[271]: 211, 169	Galloyl-*O*-hexoside
3	2.1	273	343	MS^2^[343]: 191, 169, 125	Galloyl quinic acid (isomer 1)
4	2.3	271	169	MS^2^[169]: 125	Gallic acid
5	6.7	274	343	MS^2^[343]: 191, 169, 125	Galloyl quinic acid (isomer 2)
6	7.6	276, 525	463 ^a,b^	MS^2^[463]: 301, 300, 337,315	Delphinidin-*O*-hexoside
			495 ^a^	MS^2^[495]: 343, 325, 191, 169	Digalloyl quinic acid
			483 ^a^	MS^2^[483]: 271, 331, 313, 439, 193, 169; MS^3^[271]: 211, 169	Digalloyl hexoside
7	8.8	274, 525	477 ^b^	MS^2^[477]: 315, 314	Petunidin-*O*-hexoside
8	9.7	274, 525	647 ^a,b^	MS^2^[647]: 495, 477	Petunidin-*O*-galloyl-hexoside derivative
			491 ^a,b^	MS^2^[491]: 329	Malvidin-*O*-hexoside
9	10.3	265, 356	631	MS^2^[631]: 479, 299, 317	Myricetin-*O*-galloyl-hexoside
10	11.0	260, 356	479	MS^2^[479]: 316, 317	Myricetin-*O*-hexoside (isomer 1)
11	11.2	260, 356	479	MS^2^[479]: 316, 317	Myricetin-*O*- hexoside (isomer 2)
12	11.4	265, 356	615	MS^2^[615]: 463, 301; MS^2^[463]: 179, 151	Quercetin *O*-hexoside- gallate
13	11.8	263, 356	449	MS^2^[449]: 316, 317	Myricetin-*O*-pentoside
14	12.1	261, 351	463	MS^2^[463]: 316, 317	Myricetin-*O*-deoxyhexoside (isomer 1)
15	12.2	261, 351	463	MS^2^[463]: 316, 317	Myricetin-*O*-deoxyhexoside (isomer 2)
16	12.9	265, 353	447	MS^2^[447]: 285; MS^3^[285]: 267, 257, 241	Kaempferol-*O*-hexoside
17	13.4	257, 350	447	MS^2^[447]: 301; MS^3^[301]: 179, 151	Quercetin-*O*-eoxyhexoside
18	14.5	265, 352	431 ^a^	MS^2^[431]: 271; MS^3^[271]: 211, 169	Galloyl derivative
			625 ^a^	MS^2^[625]: 479, 317	Myricetin-*O*-hexosyl-deoxyhexoside
19	14.6	273, 350	569	MS^2^[569]: 485, 317	Myricetin-*O*-galloyl ester
20	16.5	276	583	MS^2^[583]: 271, 565, 313, 211, 331; MS^3^[271]: 211, 169	Gallomyrtucommulone F
21	16.8	276	567	MS^2^[567]: 271, 313, 211, 169; MS^3^[271]: 211, 169	Gallomyrtucommulone C
22	17.6	276	467	MS^2^[467]: 271, 313, 169, 211; MS^3^[271]: 211, 169	Gallomyrtucommulone-type (isomer 1)
23	17.8	276	467	MS^2^[467]: 271, 313, 169, 211; MS^3^[271]: 211, 169	Gallomyrtucommulone-type (isomer 2)

Peak numbers correspond to those depicted in Figure 3; ^a^ co-eluted compounds in a peak fraction; ^b^ the respective [M]^+^ ions were registered in the positive mode. t_R_, retention time; λmax, wavelength of maximum absorbance.
